# Priorities for supported decision‐making with persons with dementia identified by lived experts

**DOI:** 10.1002/alz70858_096709

**Published:** 2025-12-24

**Authors:** Sussman Tamara, Xueping Ma, Oluwagbemiga Oyinlola, Diandra Serrano, Michaela Fleming, Paulette V. Hunter, Maryse Soulières, Lynn McCleary, M. Ariel Cascio, Rym Zakaria

**Affiliations:** ^1^ McGill University, Montreal, ON, Canada; ^2^ MGill University, Montreal, QC, Canada; ^3^ McGill University, Montreal, QC, Canada; ^4^ St Thomas More College, University of Saskatchewan, Saskatoon, SK, Canada; ^5^ Université de Montréal, Montreal, QC, Canada; ^6^ Brock University, St Catharines, ON, Canada; ^7^ Michigan State University, East Lansing, MI, USA; ^8^ Centre de recherche et d'expertise en gérontologie sociale, Montreal, QC, Canada

## Abstract

**Background:**

The United Nations Convention on the Rights of Persons with Disabilities asserts that all persons with disabilities have the right to receive the support they require to participate in decisions that affect them. This includes accessing support from trusted others, such as family and friends. Yet persons with dementia are often excluded from participating in decisions that matter most to them.

**Method:**

This presentation summarizes findings from the thematic analysis of two focus groups with persons with dementia (group 1, *n* = 3) and families (group 2, *n* = 8) convened as advisory group members to discuss their lived experience with supported decision‐making. The presentation also compares these priorities to the results of a scoping review of the literature (55 articles) which were shared with advisory group members to identify research priorities and gaps.

**Results:**

Our thematic analysis of deliberations with persons with dementia revealed that time and space to support participation and an emphasis on maintained capacities are critical in empowering people with dementia to express their preferences. Our analysis further suggested that pressure to make immediate decisions and overlooking familial expertise compromises inclusion for both persons with dementia and their families. Decisions regarding daily life and relocation were identified as decisional domains of high importance to persons with dementia and/or their families.

The importance of trust, adapted communication and inclusion in daily care featured prominently in our scoping review results. However, the temporal aspects of supported decision, familial exclusion, and supported decision‐making around relocation, identified as priorities by advisory group members, were relatively absent from our scoping review findings.

**Conclusion:**

Plans for the further development of this work, in light of these results will be discussed.

